# Granfluencers on TikTok: Factors linked to positive self-portrayals of older adults on social media

**DOI:** 10.1371/journal.pone.0280281

**Published:** 2023-02-07

**Authors:** Reuben Ng, Nicole Indran

**Affiliations:** 1 Lee Kuan Yew School of Public Policy, National University of Singapore, Singapore, Singapore; 2 Lloyd’s Register Foundation Institute for the Public Understanding of Risk, National University of Singapore, Singapore, Singapore; Chiang Mai University, THAILAND

## Abstract

Lately, there have been news reports on the rise of older content creators on various social media outlets. However, while journalists have picked up on this topic, scholars have been slow to accord it any attention. Our study delves into this topic and looks at how older TikTokers’ self-perceptions of aging are expressed in their videos. Specifically, we analyze the valence of the content of these videos and factors associated with older adults’ self-presentations. TikTok has only gone from strength to strength since its global launch in 2017. Even as stay-at-home orders and safe distancing protocols amid the COVID-19 pandemic have led to a dramatic increase in the consumption of media across various platforms, TikTok stands out among its rivals in terms of growth and user activity. Given its wide reach, content on TikTok has the potential to influence public opinion. We collated 1,382 videos created by TikTokers aged 60 and above with at least 100,000 followers. These videos amassed over 3.5 billion views. Following previous studies, three raters coded each video for valence (negative-neutral-positive). We found that women created twice as many videos as men. The middle-old group (ages 75–84) created more videos than the young-old and oldest-old groups. Positive videos outnumbered negative ones by 10 times. As hypothesized, themes linked to positive self-portrayals were ‘physical functioning’ (*p* < .001) and ‘social interaction’ (*p* < .001). Conversely, ‘cognitive functioning’ (*p* < .001) evidenced a negative association, controlling for age and gender of the user. This is one of the first studies on older adults’ self-portrayals of aging on TikTok. Our findings suggest that older adults tend to portray themselves positively across various domains on the application. If used purposefully, TikTok may serve as a promising platform for improving public perceptions of old age.

## Introduction

Although generally regarded as such, no longer is social media the preserve of younger people. Lately, there have been news reports on the rise of ‘granfluencers’ [1]—a portmanteau of ‘grandparent’ and ‘influencer’—or more broadly, older content creators on various social media outlets [2, 3]. However, while journalists have picked up on this topic, scholars have been slow to accord it any attention. Our study delves into this topic and looks specifically at how older TikTokers’ self-perceptions of aging are expressed in their videos.

Existing inquiry into self-perceptions of aging has been conducted primarily through surveys and questionnaires [4, 5]. We depart from these traditional methods for several reasons. First, unlike survey responses, posts on social media tend to be unsolicited, thus offering a glimpse into information regarding users’ behaviors beyond the context of the study [6]. Second, while surveys allow researchers to extract in-depth information concerning individuals’ thoughts and behaviors, responses are often anonymized, which means that participants may be more willing to disclose sensitive information [7]. In contrast, the presence of an audience on social media means that users may be motivated to curate their online personas [8], which could prompt older adults to either foreground or conceal certain aspects of their aging experiences.

From a constructionist viewpoint, age is not merely a biological fact, but a social construct with no inherent meaning beyond that which is assigned to it in specific social contexts [9]. Evidence indicates that later life is mostly conceived unfavorably, with societal views on old age having become increasingly negative over the past two centuries [10, 11]. At the same time, recent years have witnessed some emerging discourse on active and successful aging [12]. The result is therefore a dichotomous stereotyping of later life, whereby an idealized image of old age is marked by health, independence and social vitality, and conversely an undesirable version by infirmity, dependency and social decline [12].

Considerable attention has been devoted to examining how older adults are portrayed on social media. Scholars have found ageist stereotypes to be endemic on Twitter and Facebook, where users view the older cohort as cognitively and physically debilitated [13, 14]. This societal tendency to pathologize the aging process intensified during the COVID-19 crisis, when misconceptions that only older persons were affected by the virus bred slighting references to it as a ‘Boomer Remover’ [14–16].

The ways in which older adults make sense of the aging process range from person to person. Various factors—personal experiences, social interactions, stereotypes and cultural values—commingle to shape their self-perceptions of aging [17]. Many older people report feeling younger than their actual age, which a handful of scholars posit is a type of defensive mechanism to dissociate themselves from the stigma of aging [18]. Some also find that older adults display greater levels of satisfaction with aging over time [19] despite the pervasiveness of ageism [16, 19–22].

‘Impression management’ describes the process by which individuals consciously or unconsciously attempt to influence how others view them in order that they might be perceived positively [23]. People are generally aware of how they are evaluated by others and will use various tactics to elicit their desired impressions [23]. Older adults may be especially concerned with managing the way they are evaluated by others, particularly in societies where old age is wedded to concepts of frailty and incompetence [24], which many of them do not identify with [25]. Older women specifically may feel pressured to present themselves in a certain way to ameliorate the disadvantages of gendered ageism [26].

Martin and colleagues [26] discovered that self-presentational concerns among older persons were mostly in relation to physical ability—whether they appear competent and independent—and to norms regarding age-appropriate behaviors. Another study found that older adults managed others’ impressions of them by using humor to downplay health problems, by engaging in downward social comparisons to appear more active than others in the same age bracket, as well as by making reference to compliments made by third parties [27].

A number of reasons make TikTok a critical medium of study. First, the micro-video sharing platform has only gone from strength to strength since its global launch in 2017. Furthermore, even as stay-at-home orders and safe distancing protocols amid the COVID-19 pandemic have led to a dramatic increase in the consumption of media across various platforms, TikTok stands out among its rivals in terms of growth and user activity [28]. There is thus significant potential for older TikTokers to transform users’ opinions of old age. Second, although information regarding the breakdown of age profiles has not been disclosed, representatives of TikTok have stated that a sizable fraction of the 152 million new users who flocked to the application in March 2020—when the COVID-19 outbreak was declared a pandemic [29]—constitute the older demographic [28].

At present, only one study on older TikTokers has been conducted [30]. This qualitative study analyzed the ways in which older persons used TikTok as a way to engage in discourses on old age. Findings revealed that older TikTokers used the platform as a way to call out ageism, to defy age stereotypes as well as to make light of age-related vulnerabilities. To deepen these findings, we explore older TikTokers’ self-portrayals of aging across physical, cognitive and social domains.

From a conceptual perspective, this study is significant in that it is among the first to use social media data to analyze self-presentations of aging. Existing social media analyses in gerontology mostly focus on the use of social media among older adults [31, 32], as well as its concomitant social [33] and cognitive benefits [34]. Research on content creation in later life is in its incipience. Nonetheless, in addition to exploring the reasons that motivate older persons to produce their own content [35, 36], prior work has also examined their use of blogs or social media to discuss ageism [30, 37], as well as the possibility for content creation to become a pathway to hone digital skills [38]. This study augments existing survey-based techniques [4, 5, 39, 40] conventionally employed to analyze older persons’ self-perceptions of aging by exploring the conscious and unconscious ways in which older TikTokers communicate their experiences of aging on social media. The manner in which older adults portray themselves on social media is likely to have far-reaching consequences on how online audiences perceive this particular cohort as well as the aging process. Thus, from a practical perspective, this study examines the potential of TikTok in buttressing ongoing efforts to reframe aging [41–45] and ultimately to eradicate ageism.

We test three hypotheses. A previous study uncovered that older adults on TikTok actively defy stereotypes of frailty in their videos by performing dance or fitness-related challenges. We thus hypothesize that content related to ‘physical functioning’ [30] will be linked to positive self-portrayals (Hypothesis 1). Next, in line with an earlier finding that older TikTokers frequently joke about cognitive impairment [30], we hypothesize that content related to ‘cognitive functioning’ will be negatively associated with self-portrayals of aging (Hypothesis 2). Third, we hypothesize that content related to ‘social interaction’ will be linked to positive self-portrayals of aging (Hypothesis 3). Evidence has shown that prosocial behavior increases as a function of age [46, 47]. Moreover, people often use social media to project a personable version of themselves [8].

## Methods

### Dataset

Following previous studies [30, 48, 49], we created a new account on TikTok to compile the relevant videos. This was done to minimize bias since videos on TikTok are arranged based on a complex algorithm which takes into account the popularity of the post (measured by views, likes, comments and shares), the popularity of the creator (measured by followers and engagement), any previous content that was engaged with, as well as the location of the device used to access the application [49]. No content was engaged with previously to ensure a common user’s experience in navigating the application. The aforementioned collection method followed previous studies [30, 48, 49] and complied with the terms of the platform.

**Fig 1:**
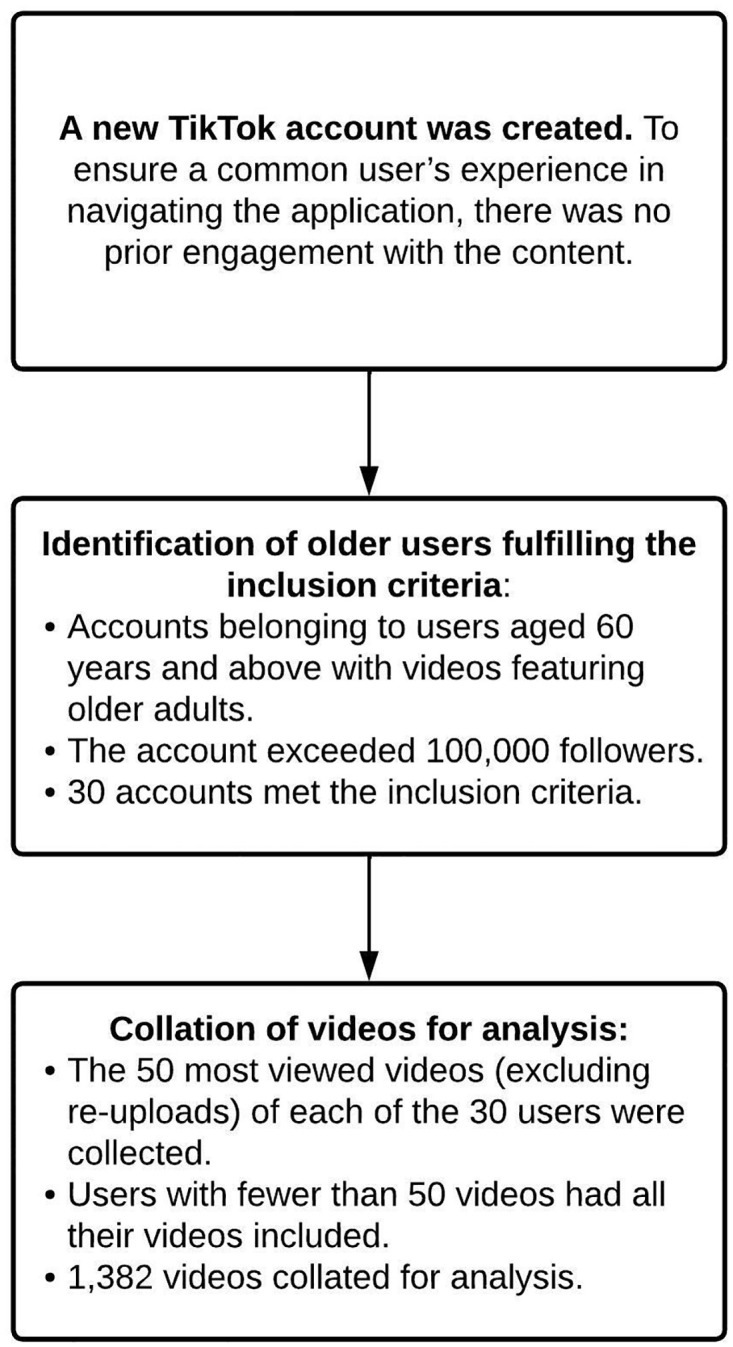
Process of collating TikTok videos.

Videos were collated with the objective of identifying accounts belonging specifically to older TikTokers. These accounts were consolidated into a database with the following inclusion criteria: (1) Account belonged to an individual aged 60 and above. The age of each content creator is public information self-declared in the user’s profile page or videos. If the user’s age was not self-declared, it was obtained from publicly available secondary sources such as news articles; (2) The follower count of the user(s) exceeded 100,000 at the time of analysis. The point of this was to ensure that the users had at least a notable degree of visibility and influence in the TikTok community. In total, 30 accounts met the inclusion criteria. As data collected were publicly available, we did not seek ethics approval or consent from the users. Nonetheless, steps were taken to protect their privacy, such as by excluding any identifying information. For each account, we collated the 50 most viewed videos excluding re-uploads. All videos were included if the account holder had fewer than 50 videos. The entire process generated 1,382 videos that received over 3.5 billion views at the time of collation. Of the 30 accounts, 28 belonged to single users. The remaining 2 accounts belonged to more than one user each. Each video had a maximum duration of one minute as mandated by the platform at the time of analysis. Fig 1 provides a flowchart of the data collection process. As the videos were publicly available, our collection and analysis complied with necessary terms and conditions.

### Coding of predictors and covariates

We referred to previous studies that explored the variables associated with self-perceptions of aging [40, 50] and adapted them to code our sample of videos. The variables are: 1) ‘physical functioning’ (presence of content related to physical health and/or functioning e.g., dancing, hip fractures); 2) ‘cognitive functioning’ (presence of content related to cognitive health e.g., dementia); 3) ‘social interaction’ (presence of content related to social interactions e.g., a protagonist cooking for his or her family). These variables were rated on a binary scale, where the presence of the attribute was rated ‘1’ and the absence of it was rated ‘0’.

With regard to covariates, demographic features of the protagonist in the video were based on publicly available information. For age, ‘0’ referred to the young-old (ages 60–74), ‘1’ referred to the middle-old (ages 75–84) and ‘2’ referred to the oldest-old (85 years and above). The video was coded ‘3’ if there were two or more protagonists in the video (e.g., a pair of siblings) whose ages did not fall into the same category. The video was coded ‘4’ (unknown) if no information regarding the user’s age was publicly available. The gender identity of each protagonist was also publicly available information with ‘0’ referring to male users and ‘1’ referring to female users. Videos were coded ‘2’ if at least one man and woman were present.

### Coding of outcome variable

The outcome variable was the valence of the content of the video, which was rated on a 3-point scale by three raters: ‘1’ was negative, ‘2’ was neutral and ‘3’ was positive. This rating scale was taken from a prior social media analysis [13] and has been established to be reliable and valid [50]. A video would be rated ‘positive’ if an older person was presented in a positive light. For example, a video of an older user performing a dance challenge would be coded as positive. Meanwhile, a video would be considered ‘negative’ if it perpetuated a negative age stereotype. A post featuring an individual making fun of dementia would be coded as negative. A video of an older protagonist narrating a day in his or her life would be coded as neutral. The inter-rater reliability of the three raters using Cronbach’s alpha was 0.89 (95% CI: 0.82, 0.93) for the scoring method.

### Analytic strategy

A linear mixed-effects model was used to test the hypotheses given the nested structure of the dataset where multiple videos belonged to each account. This model is an extension of linear regression and involves the estimation of both fixed effects and random effects. To avoid violating the independence assumption, we included random intercepts for the user accounts. All content categories rated for each video as well as demographics were included as fixed effects. All analyses were performed in R version 4.1.0.

## Results

### Descriptive statistics

Our dataset contained 1,382 videos which received a total of 3.5 billion views at the time of data collection. The mean number of views per video was 2.7 million. Most videos were created by the middle-old group (34.8%), followed by the young-old group (27.4%) and finally, the oldest-old group (21.7%). The remaining videos were created by older adults whose ages we were unable to verify using publicly available sources. Women (64.6%) created twice as many videos as men (27.3%), with a small proportion of videos featuring both men and women (8.1%). Positive/neutral videos outnumbered negative ones by 10 times. The themes linked to positive videos were ‘physical functioning’ (53.1%) and ‘social interaction’ (45.3%). The theme linked to negative videos was ‘cognitive functioning’ (15%). See Table 1 for the descriptive statistics.

**Table 1:** Description of video features and demographics of TikTok videos uploaded by older adults by valence of content ^a^.

Valence of Video Content (Outcome)	Negative		Neutral		Positive		p^b^
	*N* = 120		*N* = 424		*N* = 838		
Video Themes							
Physical Functioning	21	17.5%	9	2.1%	445	53.1%	0.000
Cognitive Functioning	18	15.0%	2	0.5%	3	0.4%	0.000
Social Interaction	19	15.8%	15	3.5%	380	45.3%	0.000
Demographics of Older Adult(s) in the Video							
Age							0.000
0—Young-old (60–74)	41	34.2%	123	29.0%	214	25.5%	
1—Middle-old (75–84)	40	33.3%	141	33.3%	300	35.8%	
2—Oldest-old (≥85)	39	32.5%	80	18.9%	181	21.6%	
3—Mix	0	0.0%	0	0.0%	50	6.0%	
4—Unknown	0	0.0%	80	18.9%	93	11.1%	
Gender							0.000
0—Man	10	8.3%	166	39.2%	201	24.0%	
1—Woman	107	89.2%	230	54.2%	556	66.3%	
2—Both present	3	2.5%	28	6.6%	81	9.7%	

^a^ Table values are N and column % for respective predictors and covariates.

^b^ P-value is for χ^2^ test.

### Mixed-effects model

As hypothesized, ‘physical functioning’ (*β* = .517, *p* < .001) and ‘social interaction’ (*β* = .384, *p* < .001) were both positively associated with valence of self-portrayals, controlling for the protagonist’s age and gender (see Table 2). Conversely, ‘cognitive functioning’ was negatively associated with valence of self-portrayals (*β* = -.740, *p* < .001). Taken together, these results support hypotheses 1 through 3. Regarding the demographic profile of the protagonists, videos with at least one man and woman (e.g., a heterosexual couple) were more likely to contain negative content (*β* = -.312, *p* = .013) compared to videos with only a male protagonist. There was no evidence of multicollinearity as the variance inflation factor (VIF) scores for all predictors and covariates fell below the conservative threshold of 5.

**Table 2:** Mixed-effects model predicting the valence of video content created by older adults on TikTok.

Model	(1)		(2)	
Demographics of Older Adult(s) in the Video (Covariates)				
Age				
Young-old (60–74 years)	Ref			
Middle-old (75–84 years)	0.07	(0.19)	0.02	(0.14)
Oldest-old (≥85 years)	-0.03	(0.21)	-0.05	(0.16)
Mix	0.52	(0.39)	0.36	(0.30)
Unknown	-0.02	(0.24)	0.00	(0.18)
Gender				
Man	Ref			
Woman	-0.07	(0.15)	0.03	(0.12)
Both present	-0.12	(0.16)	-0.31*	(0.13)
Video Themes (Predictors)				
Physical Functioning			0.52***	(0.03)
Cognitive Functioning			-0.74***	(0.11)
Social Interaction			0.38***	(0.04)
N	1382		1382	
Marginal R^2^	0.03		0.26	
Conditional R^2^	0.34		0.44	

Note: Standard errors in parentheses.

* p<0.05,

** p<0.01,

*** p<0.001.

Constant not shown.

## Discussion

Our study contributes to the limited body of research on the use of social media among older adults. The most avid content creators belonged to the middle-old category, followed by the young-old and oldest-old. Women created twice as many videos as men. Evidence shows that women are more likely to be consumers of social media and men more likely to be content creators [51]. However, some have also noted that among teenagers, girls are just as likely to upload videos online as boys [52]. Regardless, the finding that older women created twice as many videos as older men ought to be viewed as an encouraging sign given the reality of gendered ageism often confronted by older women [53]. An earlier study observed that older women were highly active on TikTok, some of them using the application to challenge stereotypes of older women as passive, acquiescent and weak [30]. Their active presence on TikTok can therefore be read as an act of defiance against a culture in which they are routinely sidelined or deemed invisible.

The differences in self-presentations across the three age groups merit greater attention. Currently, most studies on media portrayals of older adults do not disaggregate the category of older people into subpopulations. However, there is a belief that those from the young-old populace frequently aspire to partake of an active retirement [54], while those in the oldest-old group are commonly seen to be in their dotage. The fact that the middle-old group created the most content—with the bulk of the videos analyzed in this study being positive—is intriguing and may help improve societal evaluations of older adults as a whole. Although it seems intuitive that life satisfaction would decrease in older age [55], particularly as health deteriorates, current scholarship fails to demonstrate any age-related decline in life satisfaction [55]. In fact, studies have demonstrated that one’s subjective well-being may actually improve with age even amid worsening physical and cognitive health [56–58]—a situation which has been termed the ‘paradox of well-being’. This paradoxical finding may be accredited to a host of factors such as the acceptance of physical limitations, contentment with achievements in life, a more realistic assessment of personal strengths and limitations, less preoccupation with social comparison and greater emotional stability [56]. Content uploaded by the middle-old group may hence aid in rectifying misconceptions of later life as a period of distress or misery.

While discourse concerning older persons on social media has been largely negative [13, 59], our findings reveal that older adults tend to portray themselves positively. Specifically, positive videos created by older people outnumbered negative ones by ten times. In contrast to prevailing societal narratives which frequently medicalize old age as a time of physical debilitation [10, 11, 13], our results illustrate that videos regarding physical functioning were more likely to be positive. In many of these posts, older adults documented themselves engaging in various forms of physical activity such as dance routines. Although at face value, it may seem that these older personalities are simply adhering to viral trends on social media—dance challenges are after all a mainstay of TikTok—it may also reflect a desire for them to present a healthy image of themselves or to show themselves attuned to ongoing discourses on active aging. These results also corroborate scholarly claims that older adults today enjoy a better constitution than their predecessors [60], which reiterates the need for society to dispense with the myth that older persons are uniformly frail and infirm.

Although later cohorts of older persons outperform those from earlier cohorts in various aspects of cognitive performance [60], posts which contained attributes regarding cognitive functioning were more likely to be negative. As argued in an earlier study [30], this may be due to the fact that many older TikTokers make light of the cognitive vulnerabilities typically associated with advanced age, for instance by joking about dementia and memory loss. Humor has been conceptualized as a multifaceted construct comprising both adaptive and maladaptive aspects [61]. These adaptive or maladaptive components may have either salubrious or deleterious effects respectively on one’s psychological well-being [61]. With regard to humor in the context of aging, one study discovered that older adults were more sensitive to jokes where age-related susceptibilities were the source of ridicule [62]. Others have posited that older persons may employ humor as a coping mechanism, poking fun at themselves so as to transcend their status as victims in an ageist society [63, 64]. Hence, while such content may run the risk of perpetuating negative age stereotypes, it could ultimately serve as an attempt by older adults to negotiate the stigma of old age [65]. Making jokes about cognitive impairment may also function as a strategy for older adults to be more well received by the TikTok community since levity and irreverence are defining features of the platform [66].

Content related to social interaction was linked to positive valence. Although there exist stereotypes of older persons being irritable, pessimistic and intolerant, they are also often perceived as warm, sincere and good-natured [67]. Our sample of TikTokers displayed behaviors which revealed them to be highly affectionate, family-oriented and caring—qualities which may have played a role in their ability to acquire a huge follower base. Numerous studies have also observed age-related gains for traits such as agreeableness [68] and generosity [69], a finding that has been accounted for using the theory of socioemotional selectivity [70].

Our findings have important implications at the macro level. Scholars have drawn attention to the prevalence of ageism on social media [13, 59]. While older adults have become more enthusiastic about joining social media over the years [71], the percentage of this cohort active on social media remains regrettably low [72]. Their limited engagement in the online space may be a major reason why ageist content persists. Hence, there remains a pressing need to promote the inclusion of older voices on social media. Whereas in the past individuals were mere passive consumers of media, today people are able to take center stage in the production and dissemination of their own content [72]. Sustained efforts could therefore be made to encourage older persons not only to use social media, but also to actively create their own content. This may empower them to take charge of their own narratives, in turn complementing existing efforts to reframe aging [41, 42]. On a related note, it is important for older people to be included as a target audience for educational interventions regarding ageism. This will equip them with the knowledge that they need to correct misconceptions about aging as well as enable them to be more intentional about the kind of content they disseminate on social media.

Although ageism is pervasive on social media, technology can be employed as a tool to build intergenerational connection [73, 74]. Worth noting is that there are features unique to TikTok, such as the ‘duet’ function, designed specifically to facilitate interaction among users [75]. Furthermore, it has been said that older TikTokers relish the opportunity to interact with younger users on the application [2, 3] and that they enjoy creating content with their grandchildren [76]. Thus, social media should be availed as an invaluable platform for interactions between different generations. In addition to providing a healthy social mechanism for the mutual exchange of knowledge, values and skills [73], intergenerational exchanges may also enhance older persons’ sense of self-worth and younger people’s understanding towards their older contemporaries [73].

This study must be interpreted in light of certain limitations. First, our sample consisted of only thirty older adults whose portrayals of aging may not be representative of the wider older demographic. Relatedly, we did not have information about users’ educational backgrounds, religious affiliations, ethnicities and other demographic variables. Second, we were not privy to users’ motivations for creating the videos. Older adults may perceive their old age in a certain way but choose to portray it in another way. Since TikTok is a public platform, these older adults may create content as a way to gain popularity. There is even the possibility that some users are managed by media agencies or family members and do not choose the kind of content to create, in which case, the data collected would not accurately showcase their own perceptions of aging. Our findings therefore need to be supplemented by other methodologies like surveys [77–81] and big data analytics [82–90].

Despite these limitations, our findings provide a useful starting point for further exploration of issues related to older content creators. Promising areas for future research include exploring the factors which motivate older adults to create TikTok videos, the effects of TikTok usage on the well-being of older adults, the interpretation of older TikTokers’ content by younger audiences and finally, the differences between content created by older people on TikTok and that on other social media platforms.

## Conclusion

Notwithstanding its destructive effects on society, the COVID-19 crisis has fortuitously brought about a stronger presence of older adults on social media. Social media has proven itself to be a powerful tool to create awareness and lobby for social change. Our findings suggest that older adults tend to portray themselves positively on TikTok. If used purposefully, TikTok may serve as a promising platform for improving public perceptions of old age.
